# Rare complex recurrent cystic echinococcosis with multi-organ involvement after inadequate postoperative therapy: a case report

**DOI:** 10.3389/fmed.2025.1687259

**Published:** 2025-10-17

**Authors:** Yue Zhong, You Yuan, Yuting Zhang, Yu Zheng, Chunyan Chen

**Affiliations:** ^1^Operating Room, Fuyong People’s Hospital of Baoan District, Shenzhen, Guangdong, China; ^2^Department of Critical Care Medicine, Affiliated Hospital of Zunyi Medical University, Zunyi, Guizhou, China

**Keywords:** cystic echinococcosis, recurrence, multiorgan involvement, surgical treatment, multidisciplinary management, case report

## Abstract

**Background:**

Cystic echinococcosis (CE) is a globally prevalent zoonotic parasitic disease. Multi-organ recurrent CE is extremely rare, particularly in patients lacking standardized postoperative antiparasitic therapy or regular follow-up.

**Case presentation:**

We report a 29-year-old Tibetan male who presented with progressive abdominal pain, distension, and a 15-kg weight loss over 6 months. He had undergone hepatic CE cystectomy 5 years earlier but did not receive regular albendazole therapy or follow-up. Imaging revealed multiple cystic lesions in the liver, spleen, greater omentum, and pelvic cavity. More than 13 lesions, including hepatic and diaphragmatic lesions, were excised during surgery, with concurrent management of vascular and urinary tract involvement. Intraoperative blood loss was approximately 2,800 mL, which required transfusion support and intensive care monitoring.

**Results:**

The patient stayed in the intensive care unit for 7 days and spent a total of 46 days in the hospital. After surgery, albendazole therapy was resumed. Follow-up evaluations at 1, 6, and 12 months showed no signs of recurrence, and long-term follow-up is still ongoing. Due to the potential for late recurrence of cystic echinococcosis, continued long-term monitoring is recommended.

**Conclusion:**

Management of multi-organ recurrent CE requires standardized surgery, multidisciplinary collaboration, prolonged antiparasitic therapy, and strict follow-up. Ensuring patient adherence and providing health education are critical for improving long-term outcomes.

## 1 Introduction

Echinococcosis is a zoonotic parasitic disease affecting humans and animals, resulting from infection by *Echinococcus granulosus or Echinococcus multilocularis* (1, 2). Transmission primarily occurs through ingestion of food or water contaminated with parasite *eggs* (3, 4). After ingestion, the eggs hatch in the intestine, releasing oncospheres that penetrate the intestinal mucosa and disseminate via the portal venous system to various organs, where they develop into larval cysts (5). Clinically, echinococcosis is classified as cystic echinococcosis (CE) or alveolar echinococcosis (AE) according to the causative species (6).

Echinococcosis exhibits marked geographic clustering and is endemic in livestock-rearing regions worldwide, including Asia, Europe, Africa, South America, and Australia (7). In China, western pastoral regions, such as Tibet, Qinghai, and Sichuan, are highly endemic (8–10). The disease has an insidious onset and a long incubation period, with clinical symptoms typically appearing 5–10 years after infection, making early diagnosis difficult. CE commonly presents with single or multiple slowly enlarging cysts, causing symptoms primarily through mechanical compression. In contrast, AE demonstrates an infiltrative growth pattern resembling that of malignant tumors, earning it the name “parasitic cancer,” and carries a 10–15-year mortality rate of up to 90% in untreated or inadequately treated patients (11, 12).

Surgery remains the preferred radical treatment for CE; however, postoperative recurrence occurs in approximately 4–16% of cases, commonly due to intraoperative cyst rupture, postsurgical parasite dissemination, or inadequate antiparasitic therapy and follow-up (13, 14). Multiorgan postoperative recurrence due to insufficient adherence to antiparasitic treatment is rare.

Here, we report a rare case of recurrent cystic echinococcosis in a 29-year-old Tibetan herdsman who underwent hepatic cyst resection 5 years earlier but did not receive regular postoperative albendazole therapy or imaging follow-up. The recurrence was only diagnosed 5 years later when the patient presented with abdominal discomfort, reflecting critical gaps in surveillance and postoperative management. It manifested as disseminated intra-abdominal disease affecting the liver, spleen, greater omentum, abdominal wall, and pelvic cavity. Management required extensive multidisciplinary surgery, including hepatic resection with partial pericystectomy, repair of the diaphragm and pericardium, ureteral decompression, and reconstruction of the vas deferens, accompanied by massive intraoperative bleeding and intensive care support. This case is particularly noteworthy due to the unusual multi-organ dissemination, the high degree of surgical complexity, and the patient’s sociocultural background of poor adherence, which collectively distinguish it from most previously reported cases. It provides valuable insights into surgical decision-making, the role of prolonged antiparasitic therapy, the necessity of structured follow-up, and strategies to improve adherence in rural and nomadic populations.

## 2 Case presentation

### 2.1 General information

The patient was a 29-year-old unmarried Tibetan man, working as a herdsman with only a primary school education. He was 174 cm tall, weighed 74 kg, and had a body mass index (BMI) of 24.4. He had lived in a high-altitude pastoral region since childhood. Five years earlier, he underwent surgical resection of a hepatic hydatid cyst, with an uneventful postoperative recovery; however, he did not receive long-term antiparasitic therapy or regular follow-up.

The current admission was at a different hospital from the initial surgery, and the operative records from the previous procedure were unavailable. Given the patient and his family’s limited educational background and cultural context, they were unable to provide detailed information about the surgery. Based on their recollections, hepatic hydatid cyst resection had been performed; however, specifics regarding the surgical technique, potential intraoperative spillage, and use of scolicidal agents were not remembered. As these intraoperative factors are well-recognized contributors to recurrence, the lack of documentation represents a significant limitation in this case.

The patient had a clear epidemiological history of exposure to livestock, including cattle, horses, and sheepdogs. He denied any history of chronic disease, drug allergy, psychiatric illness, or hereditary disorders, and no family members had similar hydatid disease. He was hospitalized after a CT scan performed at an outside hospital revealed multiple intra-abdominal lesions 9 days before admission.

### 2.2 Clinical presentation and physical examination

Over the past 6 months, the patient experienced progressive dull-aching pain and distension in the upper and right upper abdomen, accompanied by persistent bloating, belching, and increased flatulence, which affected normal eating and daily activities. He also reported an unintentional weight loss of approximately 15 kg. The patient was alert, in a self-supported upright position, and cooperative during examination. Vital signs were as follows: temperature 36.6 °C, pulse 78 beats/min, respiratory rate 20 breaths/min, and blood pressure 118/66 mmHg. Abdominal examination revealed a soft abdomen without palpable masses, tenderness, rebound tenderness, or shifting dullness.

### 2.3 Laboratory investigations

Laboratory evaluation revealed an elevated C-reactive protein (CRP) level of 34.2 mg/L. Serological testing by enzyme-linked immunosorbent assay (ELISA) was positive for *antibodies to Echinococcus*. Other parameters, including complete blood count, coagulation profile, serum biochemistry, thyroid function tests, and tumor biomarkers, were all within normal ranges.

### 2.4 Imaging examinations

**Figure 1:**
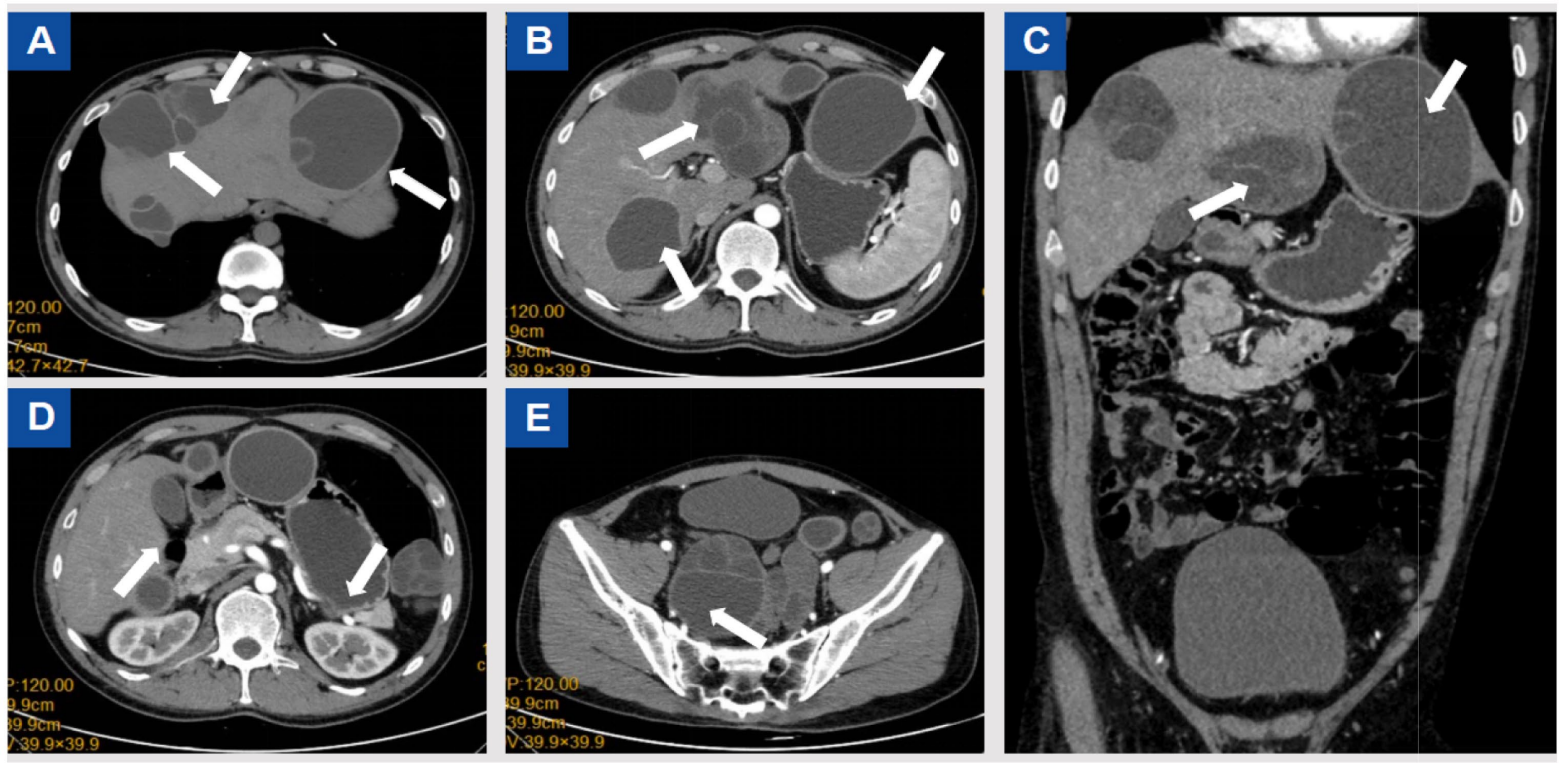
Preoperative CT scans. According to the WHO-IWGE classification, the hepatic cysts corresponded to CE2–CE3b stages, while the splenic and pelvic cysts were CE2.

Cranial CT was unremarkable. Chest and abdominal CT revealed multiple small nodules in the posterior basal segment of the left lower lung and the middle lobe of the right lung, with mild bronchiectasis and infection in the right middle lobe, as well as a small pericardial effusion. Multiple cystic lesions were observed in the liver, abdominal cavity, and pelvic cavity, some of which compressed the left ureter, causing mild hydronephrosis. The cystic lesions included localized small cyst clusters and the typical “floating membrane sign,” with several lesions extending beyond the hepatic contour. The largest left hepatic lesion measured approximately 88 × 77 mm, the right S8 lesion approximately 95 × 75 mm, the inferior splenic margin cyst approximately 40 × 76 mm, a minor lesion anterior to the greater omentum measured 23 × 28 mm, and the largest pelvic multilocular lesion measured 109 × 103 mm. All cysts demonstrated internal septations, as shown in Figure 1. Due to long-term lack of follow-up, early symptoms were atypical, resulting in delayed diagnosis. Using WHO-IWGE criteria, the main lesions were staged as follows: the largest left hepatic lesion demonstrated detached membranes consistent with CE3a; the right S8 lesion with daughter cysts and internal septations corresponded to CE3b; and the inferior splenic margin cyst and the pelvic multilocular lesions demonstrated multivesicular architecture consistent with CE2. These stage assignments informed the decision for open surgical resection combined with perioperative scolicidal measures.

### 2.5 Multidisciplinary preoperative assessment and surgical plan

Following multidisciplinary evaluation, the patient was diagnosed with disseminated recurrent cystic echinococcosis (CE) with clear indications for surgery. The lesions extended from the diaphragm to the pelvic floor, presenting considerable operative complexity and risks, including cyst rupture with potential anaphylactic shock, massive hemorrhage, and urinary system injury, as well as postoperative complications such as residual cavity infection or urinary fistula.

The planned surgical approach involved exploratory laparotomy under general anesthesia, with initial cyst decompression followed by sequential excision, prioritizing lesions compressing the ureter or at risk of rupture. External cyst excision was combined with 30% hypertonic saline for inactivation. Adequate blood products, suction devices, and emergency medications were prepared preoperatively, with ureteral stent placement if necessary. Postoperatively, the patient was transferred to the ICU for intensive monitoring and continued albendazole therapy, with regular imaging follow-up. Given the multiorgan involvement in recurrent CE, the patient was considered at high risk for adverse outcomes.

### 2.6 Surgical procedure and results

**Figure 2:**
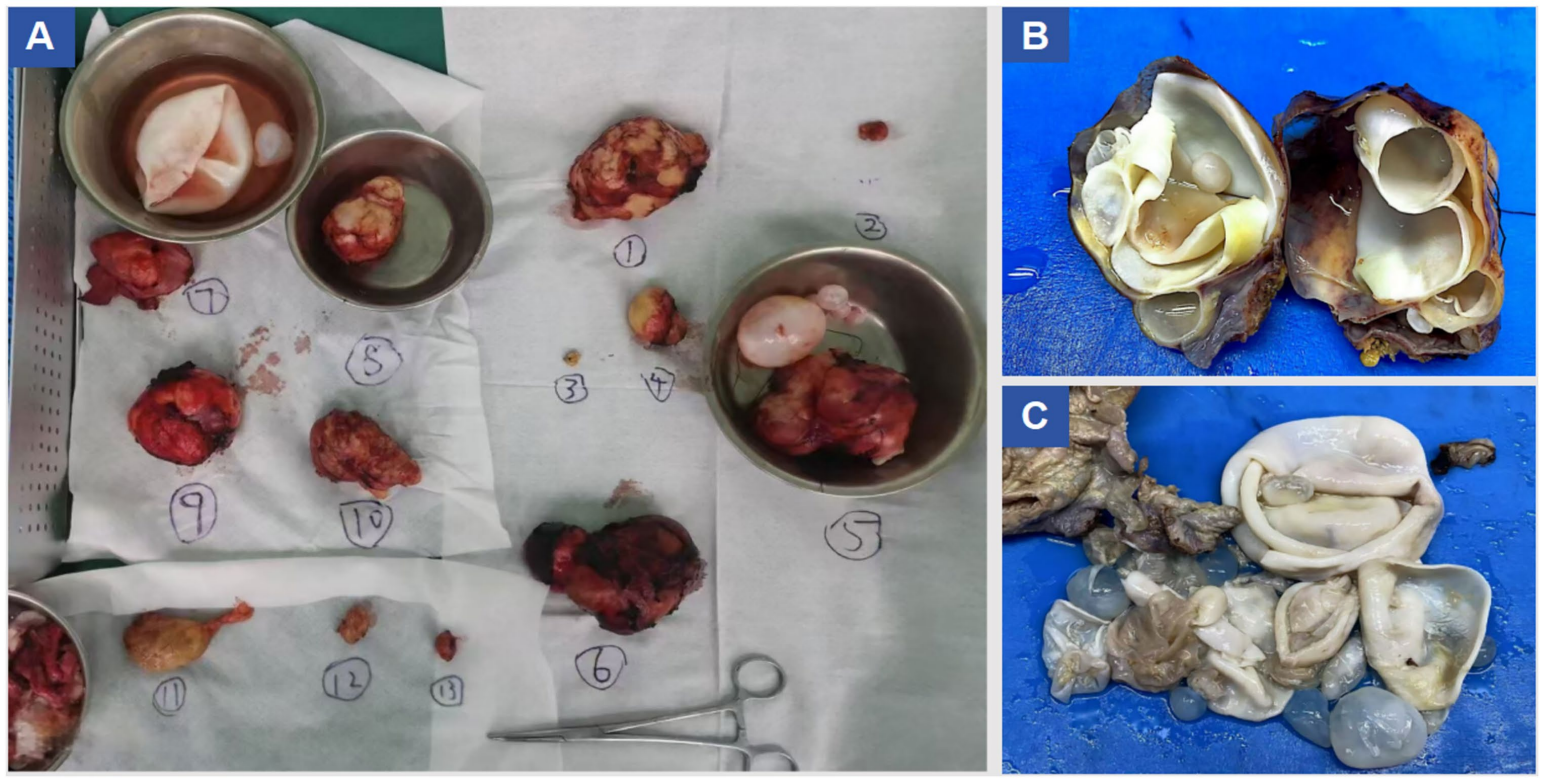
Resected specimen images.

**Figure 3:**
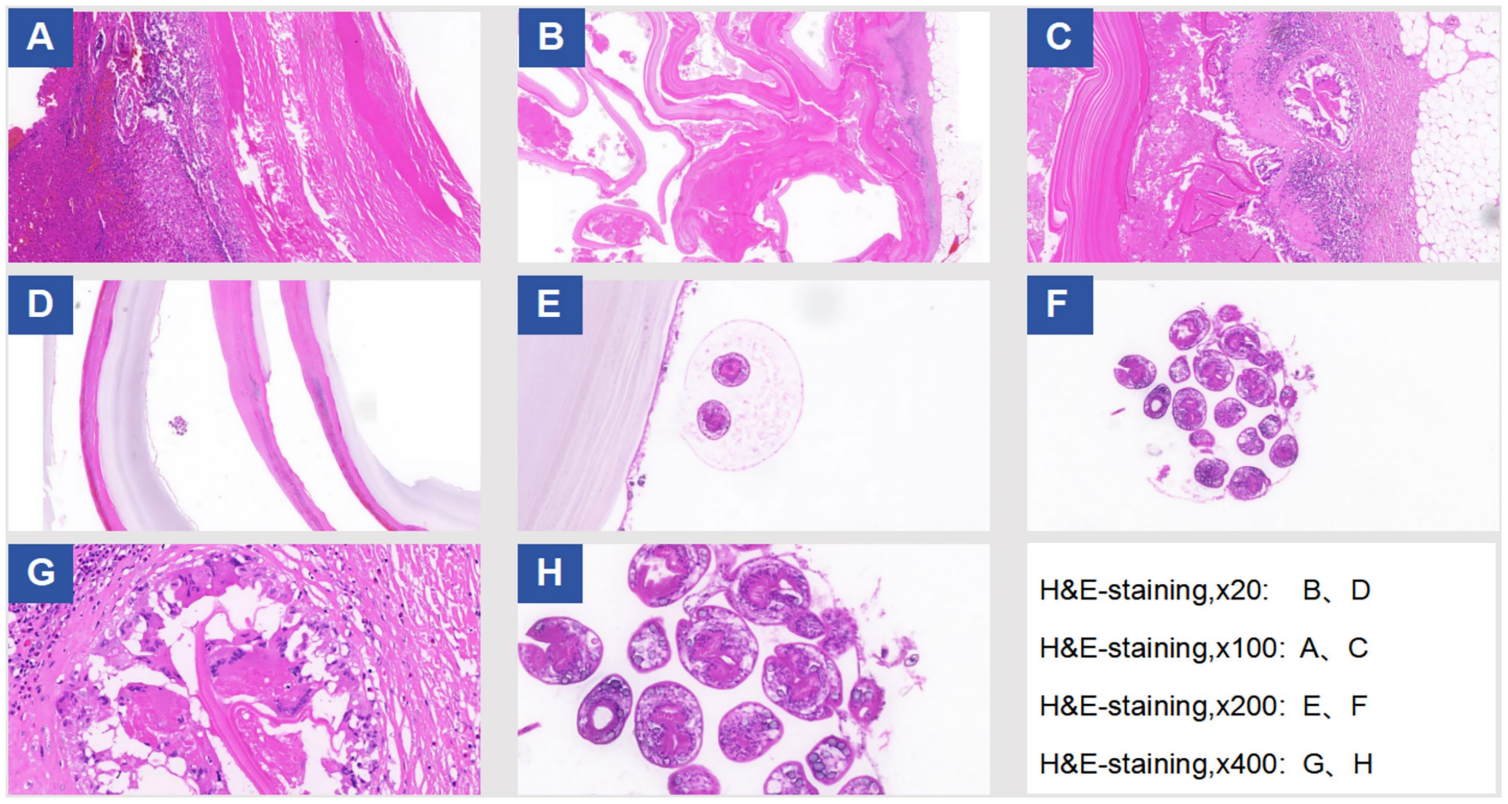
Histopathological examination results under H&E staining.

Under general anesthesia, a midline T-shaped laparotomy extending from the xiphoid process to the pubic symphysis was performed. Extensive adhesions involving the intestines, greater omentum, and abdominal wall were carefully lysed. Multiple cystic echinococcosis lesions were identified from the subdiaphragmatic space down to the pelvic cavity. To reduce intracystic tension, decompression was first conducted using a fine-gauge needle, followed by meticulous dissection along the cleavage planes between the cysts and surrounding tissues. An approximately 10 cm cyst located anterior to the stomach, together with the greater omentum, was excised while preserving the gastric wall. A roughly 4 cm lesion in the left lower abdominal wall extending into the left iliac fossa and the sigmoid mesentery was removed, and the compressed left ureter was released to restore patency. A large cyst (approximately 9.5 × 7.5 cm) situated in Couinaud segment VIII of the right hepatic lobe and protruding into the diaphragmatic dome was resected; the involved portion of the diaphragm was excised, and a partial tear of the inferior vena cava was repaired. A 9 × 6 cm cyst in the left hepatic lobe involving the pericardium was resected, followed by closure of the diaphragmatic defect. A fused lesion (approximately 10 × 6 cm) involving hepatic segments III and IV b was removed en bloc. Pelvic cysts adherent to the intestines and vas deferens were sharply dissected, with the vas deferens transected and reconstructed via end-to-end vasovasostomy. The Pringle maneuver was intermittently applied to control hepatic inflow. Estimated intraoperative blood loss was approximately 2,800 mL, and hemodynamic stability was maintained using transfusion of packed red blood cells and fresh frozen plasma. Multiple drains, including hepatic cut-surface drains, left and right subdiaphragmatic drains, pelvic drains, a drain at the foramen of Winslow, intraperitoneal drains, and a right-sided closed thoracic drainage tube—were placed to evacuate blood, fluid, and air and to monitor for postoperative complications such as hemorrhage, bile leakage, or pneumothorax. Instrument and sponge counts were confirmed correct, and the abdominal wall was closed in layers. The patient was then transferred to the intensive care unit for monitoring. A total of at least 13 lesions were completely resected during surgery, as shown in Figure 2. A summary of the surgical resection and repair procedures is provided in Table 1. Postoperative pathological examination confirmed the diagnosis of echinococcosis, as shown in Figure 3.

**Table 1:** Summary of surgical resection and repair procedures.

Location/region	Lesion size and characteristics	WHO-IWGE	Surgical procedure	Special management/complications
Previous surgical incision	1.5 × 1.2 cm firm nodule	CE4	Complete resection with electrocautery, sent for pathology	
Gastro-omental cyst (anterior to stomach)	10.7 × 7.5 cm, intact cyst wall	CE3a	Dissection with harmonic scalpel and complete cyst removal	Gastric wall preserved
Left lower abdominal wall cyst	1.0 × 0.6 cm	CE1	Excision of cyst wall and partial peritoneum	
Left iliac fossa (adjacent to sigmoid mesocolon) cyst	4.2 × 3.5 cm, compressing left ureter with proximal dilatation	CE3b	Retroperitoneal exposure, cyst dissection along ureter	Ureter released, no urinary leakage
Right liver S8 cyst	9.5 × 7.5 cm, protruding into diaphragm	CE3a	Cyst dissection along pericystic plane, resection of invaded diaphragm	Entered right thoracic cavity, diaphragmatic repair
Left hepatic lobe cyst	9.5 × 6.5 cm, invading diaphragm and pericardium	CE3b	Cyst removal via pericystic plane, partial resection of diaphragm and pericardium	Intraoperative Inferior Vena Cava rupture → immediate repair
Hepatic S3–4b fused cysts	10.5 × 6.0 cm, double cyst fusion	CE3a	Cyst resection with partial hepatic tissue removal	Stepwise hemostasis by coagulation
Inferior splenic margin cyst	7.6 × 4.0 cm	CE2	Cyst mobilization and resection	
Abdominal wall adhesions	Multiple lesions, diameter 1–2 cm	CE4	Gradual complete resection with electrocautery	
Pelvic cysts with involvement of bilateral vas deferens	Largest 10.9 × 10.3 cm, multiple, with internal septa	CE3b	Dissection and cyst removal with harmonic/electrocautery	Bilateral vas deferens transection and reconstruction (urology collaboration)

Other auxiliary procedures: Ligation of the round and falciform ligaments up to the second hepatic hilum; pre-placement of Pringle maneuver with intermittent first porta hepatis clamping (15 min occlusion/5 min release). Intraoperative bleeding and support: Total blood loss of 2,800 ml; transfusion of 15 units of packed red blood cells, 2,200 mL plasma, and 40.5 units of cryoprecipitate; patient transferred to ICU postoperatively.

### 2.7 Follow-up and outcomes

**Figure 4:**
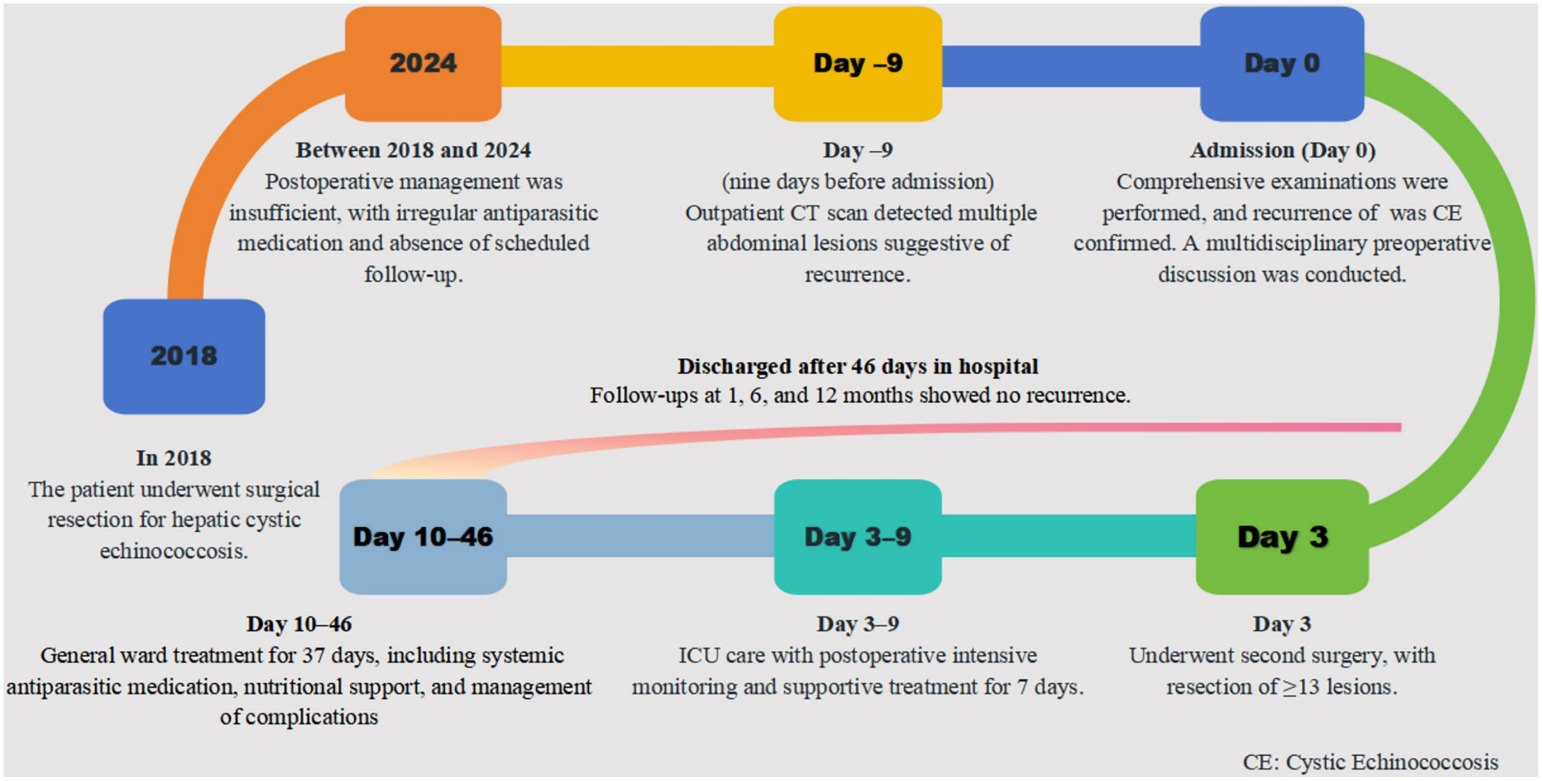
Timeline of patient case.

Postoperatively, the patient remained in the ICU for 7 days of intensive monitoring before transfer to the general ward for continued care. The total hospital stay was 46 days. Upon discharge, the patient commenced albendazole therapy and was followed up at 1, 6, and 12 months postoperatively, with no evidence of recurrence. The patient demonstrated good medication adherence, tolerated therapy well, and reported no significant adverse effects. Long-term follow-up continues, and a timeline of the patient’s treatment course is shown in Figure 4.

## 3 Discussion

Currently, the main recognized species of *Echinococcus* include *Echinococcus granulosus* (E.g.), *Echinococcus multilocularis* (E.m), *Echinococcus orligarthrus* (E.o), *Echinococcus vogeli* (E.v), and *Echinococcus shiquicus* (E.s). Among them, cystic echinococcosis (CE), caused by the larval stage of *E. granulosus*, and *alveolar echinococcosis* (AE), caused by the larval stage of *E. multilocularis*, are the most widely distributed globally (15). This case represents a rare instance of recurrent CE, with lesions involving the liver, spleen, greater omentum, and pelvic cavity, which is infrequently reported in the literature. The patient underwent hepatic cyst resection 5 years prior but did not receive standardized antiparasitic therapy or long-term follow-up, potentially resulting in residual lesions or reinfection, highlighting the need for careful long-term management and risk prevention in such cases.

### 3.1 Disease characteristics and diagnostic challenges

Echinococcosis is a zoonotic parasitic disease with a worldwide distribution. High-prevalence areas include the Mediterranean region, the Middle East, South America, Oceania, Africa, and the pastoral regions of western China, where close contact with livestock such as cattle, sheep, and dogs increases the risk of infection (2, 16–18). The liver is the most commonly affected organ, accounting for approximately 75% of cases, followed by the lungs and brain (19, 20). Early disease is often asymptomatic; however, as lesions enlarge, patients may experience discomfort in the right upper quadrant, upper abdominal fullness, or hepatomegaly. Some may present with jaundice, nausea, vomiting, or anorexia due to compression of adjacent structures, and cyst infection or rupture can lead to allergic reactions or even anaphylactic shock. Alveolar echinococcosis (AE), characterized by infiltrative growth, clinically resembles malignant tumors and often presents with hepatic masses, progressive weight loss, and hepatic dysfunction (21). If untreated, AE carries a 10-year mortality rate of up to 94%, hence its designation as “parasite cancer.”

Diagnosis requires a multidisciplinary approach. Imaging is the first-line modality, ultrasound being highly suggestive of CE, particularly when signs such as the “floating membrane” are present. AE typically requires multimodal imaging, including computed tomography (CT) for calcification and necrosis, magnetic resonance imaging (MRI) to evaluate relationships with vessels and bile ducts, and positron emission tomography/CT (PET/CT) to assess metabolic activity and detect recurrence (22–24). Serological tests, such as enzyme-linked immunosorbent assay (ELISA), indirect hemagglutination assay (IHA), and Western blot (WB), provide additional value when imaging findings are atypical (25). Epidemiological history (e.g., residence in pastoral areas, close contact with dogs or sheep) combined with pathological evidence of cyst walls, daughter cysts, protoscoleces, or hooklets is essential for definitive diagnosis. In this case, the patient’s prolonged exposure to livestock and dogs, together with characteristic clinical and radiological features, strongly suggested CE, which was ultimately confirmed pathologically as recurrent CE.

### 3.2 Treatment strategies for echinococcosis

Surgical resection remains the primary treatment for echinococcosis, particularly for isolated large cysts (26). Total pericystectomy or hepatic resection can theoretically achieve a radical cure, but the choice of surgical approach should be guided by WHO-IWGE staging and cyst characteristics. CE1–CE3a lesions, if meeting appropriate criteria, can be managed with puncture, aspiration, injection, and re-aspiration therapy (27, 28), whereas CE2–CE3b multilocular or solid-matrix cysts typically require open surgery or extended resection to remove daughter cysts and reduce intraoperative seeding (27). In recurrent or disseminated disease, radical surgery can reduce residual cavities and recurrence risk where feasible, but some lesions may not be suitable for surgery. Therefore, staged or combined approaches are often necessary, supplemented with long-term albendazole therapy (28, 29). Minimally invasive surgery and novel pharmacological agents offer additional treatment options. Nevertheless, postoperative recurrence remains a significant challenge (30, 31). To reduce recurrence risk, systemic antiparasitic therapy is recommended postoperatively to eliminate residual larvae and minimize seeding from surgical or puncture procedures (32, 33). However, long-term therapy may be associated with hepatotoxicity, myelosuppression, and other adverse effects, potentially affecting patient adherence.

### 3.3 Mechanisms of recurrence and risk factors

The mechanisms of recurrence and multiorgan involvement may include: (1) anatomical factors, with the liver acting as a “filter” via the portal vein, making it prone to larval retention; (2) iatrogenic factors, such as cyst fluid spillage during surgery or puncture leading to peritoneal implantation (13); (3) environmental factors, with continued exposure to dogs and livestock in endemic areas increasing reinfection risk (34, 35); (4) immunological factors, where impaired host immunity hinders parasite clearance and promotes recurrence and dissemination; and (5) insufficient long-term monitoring and pharmacological therapy (36).

Multi-organ intra-abdominal recurrence involving more than two sites is extremely rare (37, 38). Most reported cases are confined to the liver or present as liver–lung or liver–spleen patterns (39, 40). In this case, extensive dissemination across the peritoneum, omentum, and pelvis suggests peritoneal seeding from iatrogenic cyst fluid spillage or inadequate intraoperative containment. Meta-analyses have identified iatrogenic spillage, incomplete pericystectomy, and absence of intraoperative scolicidal application as major predictors of recurrence (13). These surgical factors may act in concert with patient-level factors, such as ongoing exposure and poor adherence to postoperative antiparasitic therapy, significantly increasing relapse risk. In the absence of detailed operative records, recurrence cannot be entirely attributed to postoperative non-adherence (41).

### 3.4 Pharmacological therapy and patient adherence

Albendazole is the primary antiparasitic agent provided free by the Chinese government for the control and prevention of echinococcosis. However, patient adherence is generally suboptimal. In a review of 582 patient records, only 22.9% of patients took albendazole regularly, 7.9% never took it, and 69.2% had irregular usage (42). Poor adherence was more common among Tibetan patients, herders, those with limited formal education, patients using the oral suspension, or those experiencing adverse effects (36, 43). A one-year imaging follow-up of 174 CE patients showed a cure rate of 5.2%, an effective rate of 32.2, 59.8% with ineffective treatment, and a 2.9% recurrence rate (42). These findings indicate that patients adhering to regular pharmacological therapy achieve significantly better outcomes than those who do not, underscoring the critical importance of improving medication adherence in enhancing therapeutic efficacy.

### 3.5 Integrated management and health education

To reduce the risk of recurrence and optimize treatment outcomes, an integrated management strategy combining surgery, pharmacotherapy, monitoring, and health education is recommended. Surgery remains the first-line treatment, particularly for isolated large cysts, where complete resection can achieve a radical cure. Albendazole, the mainstay antiparasitic agent, should be administered long-term in patients with non-calcified small cysts or multiple cysts, typically for at least 2 years and, in some cases, for a lifetime (44, 45). Regular imaging and serological follow-up are essential for the early detection of recurrence and timely adjustment of therapy (46).

Health education is particularly important in rural and nomadic populations, where adherence to albendazole therapy is often suboptimal (47). Strategies to improve long-term adherence include culturally adapted education using local languages, village-based outreach, and peer experience-sharing to raise awareness of the disease and correct drug use (48, 49). Follow-up personnel should receive systematic training, maintain standardized records, and provide flexible drug formulations tailored to patient needs (50). Mobile health tools, rural health station involvement, and continuous communication are critical to ensure uninterrupted therapy during migration (51). Additionally, reliable drug supply and strategic distribution can reduce treatment gaps and support sustained adherence (52).

## 4 Conclusion

This case reports a rare instance of recurrent cystic echinococcosis involving the liver, abdomen, and pelvic cavity. Through comprehensive multidisciplinary management, the patient achieved complete recovery without recurrence. The case underscores the importance of standardized surgical techniques, systematic management, and long-term follow-up in regions endemic for echinococcosis. Moreover, improving patient adherence to antiparasitic therapy is a key factor in enhancing treatment outcomes and reducing recurrence. Future research should investigate effective strategies to enhance medication adherence and refine comprehensive management plans, thereby further reducing recurrence rates and improving patient quality of life. This case also highlights the practical importance of strengthening primary prevention and improving patient compliance in reducing echinococcosis recurrence.

### 4.1 Limitations

The main limitation of this case is the lack of detailed information regarding the initial surgery, which limits our ability to accurately determine the causes of recurrence. Without operative reports, it is difficult to assess whether factors such as incomplete cyst removal, intraoperative spillage of cyst contents, or absence of scolicidal measures contributed to the relapse. This uncertainty complicates the interpretation of the patient’s disease course and reduces the precision of conclusions regarding risk factors for recurrence. In addition, the relatively short follow-up period of 12 months further limits the assessment of long-term outcomes, and prolonged monitoring is still required to evaluate the risk of late recurrence. During the follow-up, no significant hepatotoxicity or myelosuppression related to albendazole was observed, and medication adherence was confirmed through prescription records and patient self-reporting.

### 4.2 Patient perspective

“Before this illness, I did not understand the need for long-term follow-up or continuous medicine. After this operation and the long treatment, I now realize the importance of taking albendazole regularly and attending imaging follow-ups. I will follow the doctors’ advice and try to cooperate with the community health services to get my medicine on time. I hope to return to my work as a herdsman in the future and will do my best to avoid re-infection” (Written informed consent was obtained from the patient for inclusion of this perspective).

## Data Availability

The original contributions presented in the study are included in the article/supplementary material, further inquiries can be directed to the corresponding authors.

## References

[1] Aziz H Seda P Aswani Y Gosse MD Krishnakumari AJ Pawlik TM. Cystic echinococcosis of the liver. J Gastrointest Surg. (2025) 29:101974. doi: 10.1016/j.gassur.2025.101974, PMID: 39864780

[2] Eckert J Deplazes P. Biological, epidemiological, and clinical aspects of echinococcosis, a zoonosis of increasing concern. Clin Microbiol Rev. (2004) 17:107–35. doi: 10.1128/cmr.17.1.107-135.2004, PMID: 14726458 PMC321468

[3] Pakala T Molina M Wu GY. Hepatic Echinococcal cysts: a review. J Clin Transl Hepatol. (2016) 4:39–46. doi: 10.14218/jcth.2015.00036, PMID: 27047771 PMC4807142

[4] Pavlidis ET Galanis IN Pavlidis TE. Current considerations for the Management of Liver Echinococcosis. World J Gastroenterol. (2025) 31:103973. doi: 10.3748/wjg.v31.i10.103973, PMID: 40093668 PMC11886533

[5] Cadavid Restrepo AM Yang YR McManus DP Gray DJ Giraudoux P Barnes TS. The landscape epidemiology of echinococcoses. Infect Dis Poverty. (2016) 5:13. doi: 10.1186/s40249-016-0109-x, PMID: 26895758 PMC4759770

[6] Ito A Budke CM. The Echinococcoses in Asia: the present situation. Acta Trop. (2017) 176:11–21. doi: 10.1016/j.actatropica.2017.07.013, PMID: 28728830

[7] Grosso G Gruttadauria S Biondi A Marventano S Mistretta A. Worldwide epidemiology of liver Hydatidosis including the Mediterranean area. World J Gastroenterol. (2012) 18:1425–37. doi: 10.3748/wjg.v18.i13.1425, PMID: 22509074 PMC3319938

[8] Fu MH Wang X Han S Guan YY Bergquist R Wu WP. Advances in research on Echinococcoses epidemiology in China. Acta Trop. (2021) 219:105921. doi: 10.1016/j.actatropica.2021.105921, PMID: 33878307

[9] Wang LY Qin M Liu ZH Wu WP Xiao N Zhou XN. Prevalence and spatial distribution characteristics of human echinococcosis in China. PLoS Negl Trop Dis. (2021) 15:e0009996. doi: 10.1371/journal.pntd.0009996, PMID: 34962928 PMC8789093

[10] Ai J Zheng J Zhu C Bai Y Shi J Zhang K. Analysis and prediction of the incidence temporal trends of echinococcosis in China from 2010 to 2021. Sci Rep. (2025) 15:6423. doi: 10.1038/s41598-025-90207-9, PMID: 39984565 PMC11845582

[11] Talafuhan W Tuoheti K Lixia Y Shuang Q Yeerjiang M Aizezi G. Trends in incidence, mortality, and Dalys of cystic echinococcosis in Central Asia from 1992 to 2021: An age-period-cohort analysis. Front Public Health. (2024) 12:1504481. doi: 10.3389/fpubh.2024.1504481, PMID: 39959907 PMC11826808

[12] Wang X Dai G Li M Jia W Guo Z Lu J. Prevalence of human alveolar echinococcosis in China: a systematic review and Meta-analysis. BMC Public Health. (2020) 20:1105. doi: 10.1186/s12889-020-08989-8, PMID: 32664905 PMC7362549

[13] Alzoubi M Daradkeh S Daradka K Shattarat LN Al-Zyoud A Al-Qalqili LA. The recurrence rate after primary resection cystic echinococcosis: a Meta-analysis and systematic literature review. Asian J Surg. (2024). 48, 78–88. doi: 10.1016/j.asjsur.2024.09.038, PMID: 39343686

[14] Altin N Acar A Ergun O Kuzi S Ulusoy T Habiloğlu AD. Review of hydatid cyst cases and causes of recurrences complicating treatment, a 7-year cross-sectional study from Turkey: a single-center, retrospective observational study. Medicine. (2025) 104:e42861. doi: 10.1097/md.0000000000042861, PMID: 40550059 PMC12187273

[15] Gemmell MA Meslin FX Pawlowski ZS. WHO/OIE manual on echinococcosis in humans and animals. Paris: a public health problem of global concern World Organisation for Animal Health (2002).

[16] Lundström-Stadelmann B Rostami A Frey CF Torgerson PR Riahi SM Bagheri K. Human alveolar echinococcosis-global, regional, and National Annual Incidence and prevalence rates. Clin Microbiol Infect. (2025) 31:1139–45. doi: 10.1016/j.cmi.2025.01.034, PMID: 40054771

[17] Vuitton DA Zhou H Bresson-Hadni S Wang Q Piarroux M Raoul F. Epidemiology of alveolar echinococcosis with particular reference to China and Europe. Parasitology. (2003) 127:S87–S107. doi: 10.1017/S0031182003004153 15027607

[18] Torgerson PR Robertson LJ Enemark HL Foehr J van der Giessen JWB Kapel CMO. Source attribution of human echinococcosis: a systematic review and Meta-analysis. PLoS Negl Trop Dis. (2020) 14:e0008382. doi: 10.1371/journal.pntd.0008382, PMID: 32569309 PMC7332091

[19] Kern P Menezes da Silva A Akhan O Müllhaupt B Vizcaychipi KA Budke C. The Echinococcoses: diagnosis, clinical management and burden of disease. Adv Parasitol. (2017) 96:259–369. doi: 10.1016/bs.apar.2016.09.006, PMID: 28212790

[20] Wen H Vuitton L Tuxun T Li J Vuitton DA Zhang W. Echinococcosis: advances in the 21st century. Clin Microbiol Rev. (2019) 32:e00075-18. doi: 10.1128/cmr.00075-18, PMID: 30760475 PMC6431127

[21] Badwaik N Gharde P Shinde RK Tayade H Navandhar PS Patil M. Hydatid cyst or echinococcosis: a comprehensive review of transmission, clinical manifestations, diagnosis, and multidisciplinary treatment. Cureus. (2024) 16:e63713. doi: 10.7759/cureus.63713, PMID: 39099980 PMC11294710

[22] Yangdan CR Wang C Zhang LQ Ren B Fan HN Lu MD. Recent advances in ultrasound in the diagnosis and evaluation of the activity of hepatic alveolar echinococcosis. Parasitol Res. (2021) 120:3077–82. doi: 10.1007/s00436-021-07262-0, PMID: 34370071

[23] Liu H Xie Y An X Xu D Cai S Chu C. Advances in novel diagnostic techniques for alveolar echinococcosis. Diagnostics (Basel). (2025) 15:585. doi: 10.3390/diagnostics15050585, PMID: 40075832 PMC11898896

[24] Liu W Delabrousse É Blagosklonov O Wang J Zeng H Jiang Y. Innovation in hepatic alveolar echinococcosis imaging: best use of old tools, and necessary evaluation of new ones. Parasite. (2014) 21:74. doi: 10.1051/parasite/2014072, PMID: 25531446 PMC4273719

[25] Erganis S Sarzhanov F Al FD Cağlar K. Comparison of methods in the serologic diagnosis of cystic echinococcosis. Acta Parasitol. (2024) 69:1122–31. doi: 10.1007/s11686-024-00840-z, PMID: 38551763 PMC11182860

[26] Kamiyama T. Recent advances in surgical strategies for alveolar echinococcosis of the liver. Surg Today. (2020) 50:1360–7. doi: 10.1007/s00595-019-01922-6, PMID: 31768657

[27] Akhan O Özbay Y Ünal E Karaagaoglu E Çiftçi TT Akıncı D. Long-term results of modified catheterization technique in the treatment of Ce type 2 and 3b liver hydatid cysts. Cardiovasc Intervent Radiol. (2025) 48:503–11. doi: 10.1007/s00270-025-03976-1, PMID: 39953155 PMC11958407

[28] Balli O Balli G Cakir V Gur S Pekcevik R Tavusbay C. Percutaneous treatment of Giant cystic echinococcosis in liver: catheterization technique in patients with Ce1 and Ce3a. Cardiovasc Intervent Radiol. (2019) 42:1153–9. doi: 10.1007/s00270-019-02248-z, PMID: 31119356

[29] Bayrak M Altıntas Y. Current approaches in the surgical treatment of liver hydatid disease: single center experience. BMC Surg. (2019) 19:95. doi: 10.1186/s12893-019-0553-1, PMID: 31315619 PMC6637587

[30] Zhao ZM Yin ZZ Meng Y Jiang N Ma ZG Pan LC. Successful robotic radical resection of hepatic echinococcosis located in Posterosuperior liver segments. World J Gastroenterol. (2020) 26:2831–8. doi: 10.3748/wjg.v26.i21.2831, PMID: 32550758 PMC7284188

[31] Denzinger M Nasir N Steinkraus K Michalski C Hüttner FJ Traub B. Treatment concepts for hepatic echinococcosis. Chirurgie (Heidelb). (2023) 94:560–70. doi: 10.1007/s00104-023-01825-w, PMID: 36853342

[32] Chiodini PL. Medical Management of Cystic Echinococcosis. Curr Opin Infect Dis. (2023) 36:303–7. doi: 10.1097/qco.0000000000000947, PMID: 37593991

[33] Kuehn R Uchiumi LJ Tamarozzi F. Treatment of uncomplicated hepatic cystic echinococcosis (hydatid disease). Cochrane Database Syst Rev. (2024) 7:Cd015573. doi: 10.1002/14651858.Cd015573 38994714 PMC11240857

[34] Wang Q Wang Y Luo Z Liao S Yu W Zhang G. Settlement characteristics and transmission of echinococcosis: a cross-sectional study in nomadic communities on the Qinghai-Tibet plateau, China. Infect Dis Poverty. (2025) 14:47. doi: 10.1186/s40249-025-01316-6, PMID: 40506786 PMC12160359

[35] Schantz PM Wang H Qiu J Liu FJ Saito E Emshoff A. Echinococcosis on the Tibetan plateau: prevalence and risk factors for cystic and alveolar echinococcosis in Tibetan populations in Qinghai Province, China. Parasitology. (2003) 127:S109–20. doi: 10.1017/S0031182003004165, PMID: 15027608

[36] Wang LY Qin M Gavotte L Wu WP Cheng X Lei JX. Societal drivers of human echinococcosis in China. Parasit Vectors. (2022) 15:385. doi: 10.1186/s13071-022-05480-8, PMID: 36271415 PMC9587573

[37] Yan L Chu Z Yang J Zhang Y Liu G Lei Z. Multiple cystic echinococcosis in abdominal and pelvic cavity treated by surgery with a 4-year follow-up: a case report. Front Med. (2024) 11:1276850. doi: 10.3389/fmed.2024.1276850, PMID: 38304097 PMC10830638

[38] Malekpour Alamdari N Ansari I Karimian M Babakhani E Hatami S Mohammadsadeghi P. Peritoneal hydatid cyst mimicking peritoneal seeding; a case report. Iran J Pathol. (2025) 20:330–4. doi: 10.30699/ijp.2025.2056341.3430, PMID: 40746932 PMC12308191

[39] Shahriarirad R Erfani A Eskandarisani M Rastegarian M Taghizadeh H Sarkari B. Human cystic echinococcosis in Southwest Iran: a 15-year retrospective epidemiological study of hospitalized cases. Trop Med Health. (2020) 48:49. doi: 10.1186/s41182-020-00238-3, PMID: 32577086 PMC7304208

[40] Shahriarirad R Erfani A Eskandarisani M Rastegarian M Sarkari B. Uncommon locations of cystic echinococcosis: a report of 46 cases from southern Iran. Surg Res Pract. (2020) 2020:1–6. doi: 10.1155/2020/2061045, PMID: 33015320 PMC7520003

[41] Velasco-Tirado V Romero-Alegría Á Belhassen-García M Alonso-Sardón M Esteban-Velasco C López-Bernús A. Recurrence of cystic echinococcosis in an endemic area: a retrospective study. BMC Infect Dis. (2017) 17:455. doi: 10.1186/s12879-017-2556-9, PMID: 28655301 PMC5488421

[42] Qin M Yang G Yan J Wang L Feng Y Wang D. Assessment of compliance and therapeutic efficacy of Albendazole treatment in Chinese patients with echinococcosis. Infect Dis Poverty. (2024) 13:98. doi: 10.1186/s40249-024-01268-3, PMID: 39707489 PMC11662814

[43] Li S Chen J He Y Deng Y Chen J Fang W. Clinical features, radiological characteristics, and outcomes of patients with intracranial alveolar echinococcosis: a case series from Tibetan areas of Sichuan Province, China. Front Neurol. (2020) 11:537565. doi: 10.3389/fneur.2020.537565, PMID: 33519658 PMC7843382

[44] Dehkordi AB Sanei B Yousefi M Sharafi SM Safarnezhad F Jafari R. Albendazole and treatment of hydatid cyst: review of the literature. Infect Disord Drug Targets. (2019) 19:101–4. doi: 10.2174/1871526518666180629134511 29956639

[45] Mahmoodi S Ebrahimian M Mirhashemi SH Soori M Rashnoo F Oshidari B. A 20 years retrospective descriptive study of human cystic echinococcosis and the role of Albendazole concurrent with surgical treatment: 2001-2021. Iran J Parasitol. (2023) 18:100–6. doi: 10.18502/ijpa.v18i1.12386, PMID: 37197074 PMC10183456

[46] Li T Ito A Pengcuo R Sako Y Chen X Qiu D. Post-treatment follow-up study of abdominal cystic echinococcosis in Tibetan communities of Northwest Sichuan Province, China. PLoS Negl Trop Dis. (2011) 5:e1364. doi: 10.1371/journal.pntd.0001364, PMID: 22039558 PMC3201905

[47] Zhang T Li B Liu Y Liu S. Risk factors associated with echinococcosis in the general Chinese population: a Meta-analysis and systematic review. Front Public Health. (2022) 10:821265. doi: 10.3389/fpubh.2022.821265, PMID: 35655451 PMC9152270

[48] Enriquez M Conn VS. Peers as facilitators of medication adherence interventions: a review. J Prim Care Community Health. (2016) 7:44–55. doi: 10.1177/2150131915601794, PMID: 26303976 PMC5695224

[49] Arshad MF Abbas I Porcu F Ricci A Gaglio G Brianti E. Breaking the cycle of parasitic diseases with edutainment: the intersection of entertainment and education. PLoS Negl Trop Dis. (2025) 19:e0013072. doi: 10.1371/journal.pntd.0013072, PMID: 40435280 PMC12119011

[50] Yin Z Lesser J Paiva KA Zapata J Jr Moreno-Vasquez A Grigsby TJ. Using Mobile health tools to engage rural underserved individuals in a diabetes education program in South Texas: feasibility study. JMIR Mhealth Uhealth. (2020) 8:e16683. doi: 10.2196/16683, PMID: 32207694 PMC7139426

[51] Wang L Wang Z Qin M Lei J Cheng X Yan J. A regressive analysis of the Main environmental risk factors of human echinococcosis in 370 counties in China. PLoS Negl Trop Dis. (2024) 18:e0012131. doi: 10.1371/journal.pntd.0012131, PMID: 38743784 PMC11125469

[52] Qian MB Abela-Ridder B Wu WP Zhou XN. Combating echinococcosis in China: strengthening the Research and Development. Infect Dis Poverty. (2017) 6:161. doi: 10.1186/s40249-017-0374-3, PMID: 29157312 PMC5697071

